# Associations between attention deficit hyperactivity and internet gaming disorder symptoms: Is there consistency across types of symptoms, gender and countries?

**DOI:** 10.1016/j.abrep.2018.100158

**Published:** 2019-01-02

**Authors:** Vasilis Stavropoulos, Baxter L.M. Adams, Charlotte L. Beard, Emma Dumble, Steven Trawley, Rapson Gomez, Halley M. Pontes

**Affiliations:** a Cairnmillar Institute, Camberwell, Australia; b University of Tasmania, Sandy Bay, Australia; c Palo Alto University, Palo Alto, United States of America; d Federation University, Ballarat, Australia; e Nottingham Trent University, Department of Psychology, International Gaming Research Unit, Nottingham, United Kingdom

**Keywords:** Internet gaming disorder, Emergent adults, Massively multiplayer online games, Attention deficit hyperactivity, gender, culture

## Abstract

**Background:**

Videogame addiction has been suggested as a tentative disorder in 2013 by the American Psychiatric Association (APA) and was recently officially recognized as a mental health disorder by the World Health Organization (WHO). Although a few studies have identified attention deficit and hyperactivity disorder (ADHD) as a key risk factor for Internet Gaming Disorder (IGD), the interplay between ADHD and IGD symptoms with gender differences across cultures remains to be further examined.

**Objective:**

This study examined the moderating effects of gender in the association between ADHD and IGD across two nations.

**Method:**

A cross-sectional online survey was developed to recruit 164 Australian (M_age_ = 23.01, *SD* = 3.35, Min_age_ = 18, Max_age_ = 31, Males *n* = 121, 73.80%) and 457 U.S.-North American (M_age_ = 25.25 years, *SD* = 2.76, Min_age_ = 18 years, Max_age_ = 29 years, Males = 265, 57.98%) Massively Multiplayer Online (MMO) players aged between 18 and 29 years.

**Results:**

The hierarchical linear regression, moderation and moderated moderation analyses revealed that participants presenting greater inattention and hyperactivity symptoms exhibited higher levels of IGD-related behaviors in the two samples. Moreover, these associations differed across genders between the two countries. Specifically, more hyperactive-impulsive, as well as inattentive males in the USA presented higher levels of disordered gaming.

**Conclusion:**

The results highlight the need for more cross-cultural and symptom-focused research in the broader IGD field.

## Highlights

• Internet Gaming Disorder (IGD) and Attention Deficit Hyperactivity Disorder (ADHD) have been associated.
• A combined sample of Australian and American Gamers was examined.
• The moderating effects of gender and country of origin were assessed regarding the ADHD-IGD associations.
• American Males gamers presenting with ADHD were at higher IGD risk.

## 1 Introduction

Videogames have changed dramatically since they were first introduced in the 1970s, with improvements in network performance and computing power facilitating the development of highly interactive multiplayer games (Griffiths, Davies, & Chappell, 2003). In particular, the introduction of Massively Multiplayer Online (MMO) games has enabled a larger number of individuals to play the same game within the same session without geographical constraints (Pontes & Griffiths, 2014).

Alongside the increased popularity of online gaming, research examining its potential effects has grown exponentially (Burleigh, Stavropoulos, Liew, Adams, & Griffiths, 2018; Liew, Stavropoulos, Adams, Burleigh, & Griffiths, 2018; Stavropoulos, Burleigh, Beard, Gomez, & Griffiths, 2018). Indeed, online and offline games have been shown to provide a number of significant benefits and positive effects for players (Anderson, Steen, & Stavropoulos, 2017; Jones, Scholes, Johnson, Katsikitis, & Carras, 2014). More specifically, moderate game play (ranging from 7 to 10 h a week) has been linked to higher emotional stability, reduced stress levels, increased social interactions, increased happiness, life satisfaction and enhanced sense of achievement, as well as greater psychological resilience in general (Anderson et al., 2017; Jones et al., 2014). Although playing videogames is considered, for the most part, a healthy activity capable of producing beneficial effects to gamers, there is mounting evidence suggesting that excessive and addictive gaming is associated with significant health-related and behavioral drawbacks (Gomez, Stavropoulos, Beard, & Pontes, 2018; Pontes, Stavropoulos, & Griffiths, 2017; Stavropoulos et al., 2018). Videogame addiction has been systematically linked to aggressive/oppositional behavior, maladaptive coping strategies, decreased academic achievement and performance, as well as sacrificing hobbies, sleep and work (Beard, Haas, Wickham, & Stavropoulos, 2017; Ferguson, Coulson, & Barnett, 2011; Kuss & Griffiths, 2012; Lehenbauer-Baum & Fohringer, 2015).

Notwithstanding the large number of studies conducted on videogame addiction, its clinical course, phenomenology and prevalence rates across different nations remains unclear due to several methodological hindrances, such as heterogeneity in the conceptual framework used to define the phenomenon and inconsistencies surrounding its psychometric assessment across different studies (Pontes & Griffiths, 2014; Stavropoulos, Beard, et al., 2018). Despite these issues, an emerging consensus exists among scholars indicating that videogame addiction constitutes a *bona fide* addiction negatively affecting a minority of gamers (Pontes, 2018).

In order to contribute to this emerging and rapidly evolving field of research, and given its interest on internet games, the present study adopts the conceptualization of Internet Gaming Disorder (IGD) (American Psychiatric Association (APA), 2013), as a globally unifying basis for future clinical and research endeavors in an area identified as requiring further investigation (APA, 2013). Accordingly, IGD, as a tentative disorder, has been operationally defined as the persistent and recurrent engagement with videogames, often with other players, leading to clinically significant impairment or distress within a 12-month period (APA, 2013). The APA suggests that disordered gaming is reflected by the endorsement of at least five of the following nine diagnostic criteria: (1) preoccupation with videogames; (2) withdrawal symptoms when gaming is taken away; (3) tolerance, resulting in the need to spend increasing amounts of time engaged in videogames; (4) unsuccessful attempts to control participation in videogames; (5) loss of interest in previous hobbies and entertainment as a result of, and with the exception of, videogames; (6) continued excessive use of video games despite knowledge of psychosocial problems; (7) deceiving family members, therapists, or others regarding the amount of gaming; (8) use of videogames to escape or relieve negative moods; and (9) jeopardizing or losing a significant relationship, job, or education or career opportunity because of participation in videogames. Arguably, the nine diagnostic criteria proposed by the APA show significant conceptual overlaps with existing operational definitions for substance use disorders (i.e., mood modification and tolerance; Kuss & Griffiths, 2012).

In this context, variations considering IGD vulnerability have been illustrated involving specific developmental stages (e.g., adolescents and emergent adults) and game genres (e.g., MMOs), as well as individual (e.g., gender, psychopathology, academic disposition, and personal attributes) and contextual characteristics (e.g., parenting and family contexts, peer associations and classroom environment; Anderson et al., 2017; Kuss & Griffiths, 2012; Pontes & Griffiths, 2014; Stetina, Kothgassner, Lehenbauer, & Kryspin-Exner, 2011). Nevertheless, a specific focus on IGD risk and protective factors has been recommended (APA, 2013), while cultural differences both in the clinical presentation and the contributing IGD factors have been assumed (Anderson et al., 2017; Pontes et al., 2017; Stavropoulos, Beard, et al., 2018).

### 1.1 IGD in emergent adulthood

A sizeable amount of research on IGD has been conducted on children and adolescents (Andreassen et al., 2016; Arnett, Žukauskienė, & Sugimura, 2014; Stavropoulos, Kuss, Griffiths, Wilson, & Motti-Stefanidi, 2017). However, Pontes and Griffiths (2014) identified that emergent adults appear to be more regular gamers than the stereotypically believed adolescents and this has been further reinforced by more recent studies (Adams et al., 2018; Burleigh et al., 2018; Liew et al., 2018). Emergent adulthood (18–29 years) is traditionally characterized by increased independence from parents/guardians, less responsibilities than older adults and intense identity and world-related exploration (Arnett, 2000; Arnett, 2015; Sussman & Arnett, 2014). According to Sussman and Arnett (2014), individuals are particularly susceptible to the development of addictive disorders during emergent adulthood due to the combination of lower inhibition and higher accessibility to addictive stimuli. Simultaneously, emergent adulthood has been reported as a developmental stage associated with a peak in internet use and involvement with videogames (Kuss, Griffiths, & Pontes, 2017; Stavropoulos, Burleigh, et al., 2018). Therefore, scrutinizing key risk and protective factors for IGD within this developmental stage is imperative. Based on this rationale, the present study aims to bridge this gap by examining the effects of specific risk factors during emergent adulthood (i.e., attention deficit and hyperactivity disorder (ADHD) symptoms and gender), whilst concurrently emphasizing on higher IGD risk game genres using two different national samples of gamers from Australia and the United States of America (USA) to capture potential country-related variations.

### 1.2 IGD and game genre

There is a wide range of game genres, these can include racing, shooting, puzzle, fighting, simulation, and role-playing games, available both in single player and/or multiplayer formats (Anderson et al., 2017; Hu, Stavropoulos, Anderson, Scerri, & Collard, 2018; Scerri, Anderson, Stavropoulos, & Hu, 2018). Recent studies have shown that IGD susceptibility appears to vary across these game genres, with MMO games consistently corresponding with a higher risk due to its unique structural characteristics (Kuss & Griffiths, 2012; Stavropoulos, Kuss, et al., 2017; Stavropoulos, Wilson, Kuss, Griffiths, & Gentile, 2017; Stetina et al., 2011). In general, MMO games are typically made up of virtual worlds that embrace large numbers of gamers worldwide, while evolving in real time (i.e., independent of the ongoing participation of individual gamers) and in relation to the accomplishment of progressively higher and often collaborative goals and challenges (Griffiths, Davies, & Chappell, 2004; Kuss & Griffiths, 2012). Typically, MMOs require the creation and development of an avatar (i.e., a character through which the player is represented) in the virtual world (Kuss & Griffiths, 2012). Using an avatar has been shown to enhance game absorption and immersion, thus increasing IGD risk (Burleigh et al., 2018; Liew et al., 2018).

Several MMO features, such as social interaction, competition, completing progressively more challenging quests and achievements, novelty, and enabling emotional management (i.e. venting aggressive and anti-social feelings) have been highlighted as potential risk factors for IGD (Adams et al., 2018; Burleigh et al., 2018; Hussain, Williams, & Griffiths, 2015; Liew et al., 2018; Tone, Zhao, & Yan, 2014). Accordingly, such features may prolong game sessions by reinforcing the levels of immersion within the virtual world and the game actions (Alexandraki, Stavropoulos, Burleigh, King, & Griffiths, 2018; Andreassen et al., 2016; Stetina et al., 2011). Therefore, this study will focus primarily on MMOs, while examining the effects of ADHD symptoms and gender on IGD behaviors, as well the potential variations in these associations between gamers from Australia and the USA.

### 1.3 IGD & ADHD

Several behavioral problems, risky behaviors and psychiatric disorders have been consistently associated with IGD symptoms in both cross-sectional and longitudinal studies (Anderson et al., 2017). Some of these factors can include personality traits, contextual factors involving the classroom, living with parents and behavioral inclinations such as hostility and social withdrawal (Stavropoulos et al., 2017; Stavropoulos et al., 2018; Stavropoulos et al., 2018). Moreover, the presence of ADHD symptoms has been shown to increase the likelihood of a gamer exhibiting IGD symptoms (Ferguson et al., 2011; Lemmens, Valkenburg, & Peter, 2011; Rikkers, Lawrence, Hafekost, & Zubrick, 2016; Vadlin, Åslund, Hellström, & Nilsson, 2016). The APA defines ADHD as a “persistent pattern of inattention and/or hyperactivity-impulsivity that interferes with functioning or development” (APA, 2013, pg. 61). According to the APA, inattention refers to the individual's difficulty/inability to maintain concentration, tendency to wander off task, lack of persistence, and difficulties sustaining focus (APA, 2013). Furthermore, hyperactivity relates to excessive motor activity during times when it is inappropriate (e.g., during meetings). Impulsivity describes the individual's tendency to act hastily in the moment without forethought (APA, 2013). The relationship between ADHD behaviors and IGD has been demonstrated both in observational (e.g., Hyun et al., 2015) and pharmacological research examining the effectiveness of traditional ADHD medications (i.e., methylphenidate and atomoxetine) in reducing IGD symptoms (Park, Lee, Sohn, & Han, 2016; Weinstein & Weizman, 2012).

In that context IGD has been viewed as both a disorder that may be secondary to ADHD or one with a reciprocal relationship with ADHD on the basis of reward seeking gratification (i.e., with one precipitating-perpetuating the other and vice versa; Vadlin et al., 2016). Accordingly, some studies have assumed that ADHD behaviors precede IGD, while others reveal that IGD behaviors perpetuate ADHD symptoms (Hyun et al., 2015; Vadlin et al., 2016). Presumably, there is no single path for primary and secondary dysfunctions and that, as with many mental health disorders, IGD may be bi-directionally associated with ADHD (Stavropoulos, Gentile, & Motti-Stefanidi, 2016; Vadlin et al., 2016). Interestingly, online games offer many different forms of in-game reward related reinforcement (Thorens, Wullschleger, Khan, Achab, & Zullino, 2012), which have been associated with response accuracy and sustained attention in children diagnosed with ADHD (Prins, Dovis, Ponsioen, ten Brink, & van der Oord, 2011). Indeed, Weinstein and Weizman (2012) discussed a possibility of a subtype of gamers that exhibit high reward dependence and are sensitive to in-game rewards due to dopamine deficiency. This hypothesis is strengthened by findings indicating a parallel increase between ADHD and IGD symptoms, as well as a significant proportion of IGD diagnosed individuals presenting with a comorbid ADHD diagnosis (Vadlin et al., 2016; Weinstein & Weizman, 2012).

In the same vein, ADHD has also been shown to be prevalent among individuals experiencing other addictive disorders (Kuss et al., 2017) including behavioral addictions, such as gambling disorder (Fatséas et al., 2016). Several arguments have been proposed to explain this association, including that excessive gaming is a means of self-medication for individuals diagnosed with ADHD, through providing an escape pathway from real-life problems (Weinstein & Weizman, 2012). Nevertheless, despite the differentiations considering ADHD manifestations (e.g. inattention vs hyperactivity-impulsivity), and extant findings regarding the potential links between ADHD and IGD association, there is a dearth of research examining separately inattention and hyperactivity-impulsivity as distinct typologies in relation to IGD. Instead, studies have primarily focused on the effect of impulsivity on IGD (Yen et al., 2017). In particular, adolescents and young adults with IGD have been shown to present with higher scores on scales of impulsivity (Ding et al., 2014; Yen et al., 2017). Additionally, as loss of control is an IGD criterion, it has been suggested that higher impulsivity may result in higher IGD susceptibility (Yen et al., 2017). Considering cultural specificities of Australia (due to its relevance to the present study), problematic internet or electronic gaming behaviors among children have been linked to hyperactivity problems (Rikkers et al., 2016).

In that line, investigating the differential associations of IGD with inattention and hyperactivity-impulsivity can be particularly important as it aligns with studies supporting their varying associations with other forms of behaviors over the course of development (Merrell, Sayal, Tymms, & Kasim, 2017). Interestingly, a study by Yen, Yen, Chen, Tang, and Ko (2009) found that inattention was a greater risk factor for excessive internet use compared to impulsivity among college students. They argued that internet activities offering immediate response and reward (such as gaming) could alleviate the boredom interwoven with inattention (Yen et al., 2009). Consequently, the present study aims to contribute to the literature by examining the IGD risk effects of hyperactivity-impulsivity and inattention separately, and in relation to gender, in the IGD high risk population of emergent adults MMO gamers, whilst concurrently assessing the cross-cultural effects between Australia and the USA.

### 1.4 The role of gender

Variations could be assumed considering the associations between attention deficit and/or hyperactivity-impulsivity and IGD according to gender (Bryce & Rutter, 2003; Ko, Yen, Chen, Chen, & Yen, 2005). Internet gaming typically includes content, more directed to males, and females have been reported to play fewer games and for a shorter time and to experience lower online ‘flow’ (Bryce & Rutter, 2003; Stavropoulos, Alexandraki, & Motti-Stefanidi, 2013b; Stavropoulos, Griffiths, et al., 2018). Gaming motivation aspects have been suggested to interfere with these differences (Ko et al., 2005). In general, females appear to use the internet differently, spending more time on social media than gaming (Cotten & Jelenewicz, 2006; Dufour et al., 2016; Fortson, Scotti, Chen, Malone, & Del Ben, 2007). Not surprisingly, males have consistently reported significantly more problems with excessive gaming than females (Desai, Krishnan-Sarin, Cavallo, & Potenza, 2010).

Similarly, considering ADHD symptoms, females have been found to predominately experience inattention, while males typically demonstrate more externalized behaviors, more consistent with hyperactivity-impulsivity symptoms (Abikoff et al., 2002; Biederman et al., 2002; Bozkurt, Coskun, Ayaydin, Adak, & Zoroglu, 2013; Hyun et al., 2015). Interestingly, the association between ADHD and IGD has been revealed to be stronger in females (Yen et al., 2009). Furthermore, female gamers are more likely to report poorer mental health than males (Ciarrochi et al., 2016). Therefore, it can be reasonably assumed that males and females experiencing different types of ADHD symptoms may differ considering their level of IGD risk. For instance, given that males have been found to typically experience more hyperactivity-impulsivity symptoms than females, which have been linked to higher IGD risk, males in this particular group may be more at risk of IGD than their female counterparts. To advance the available knowledge, this study will independently explore whether and how gender moderates the links between inattentive and hyperactive-impulsive symptoms and IGD across MMO gamers from Australia and the USA.

### 1.5 Country/culture related effects: USA and Australia

Despite the significance of cultural factors for addictions (Griffiths, 2005) and absorbing internet use in particular (Stavropoulos, Wilson, et al., 2017), these have been under-explored in regards to IGD (Pontes, 2018; Stavropoulos, Anderson, et al., 2018; Stavropoulos, Griffiths, et al., 2018). Specifically, cultural differences in relation to the presentation of addictions (i.e., prevalence, incidence, and clinical manifestations), as well as variations considering their associated risk factors have been suggested (Landrine, Klonoff, & Brown-Collins, 1992; Stavropoulos, Anderson, et al., 2018; Wilsnack, Vogeltanz, Wilsnack, & Harris, 2000). In that line, differences between Australia and the USA considering the interplay between different types of ADHD symptoms, gender and IGD behaviors are emphasized here (Burleigh et al., 2018; Liew et al., 2018; Stavropoulos, Burleigh, et al., 2018). These national populations of MMO gamers were selected as the USA and Australia: a) are ranked within the top 15 consumers of internet games (Global Games Market Report, 2016) and b) have generated significant IGD research and treatment efforts (Kaptsis, King, Delfabbro, & Gradisar, 2016; King, Kaptsis, Delfabbro, & Gradisar, 2016). Furthermore, Australia and the USA are assumed to differ considering cultural dimensions such as “vertical” and “horizontal” individualism, which have been associated with potential differences in vulnerability to specific forms of psychopathology, (Clemens, Begum, Harper, Whitty, & Scuffham, 2014; Singelis, Triandis, Bhawuk, & Gelfand, 1995; Stavropoulos, Alexandraki, & Motti-Stefanidi, 2013a; Stavropoulos, Anderson, et al., 2018). From a cultural perspective “individualism” signifies personal interests and needs as more important compared to those of the group(s) that one might belong to (collectivism signifies group needs as more important; Lee & Wohn, 2012). Furthermore, the notion of “verticality” in “vertical individualism” describes the simultaneous presence of inequality (i.e., inequality in social benefits and access to services as well as the perceived social hierarchy) alongside with the individualistic tendencies (while the notion of “horizontal” individualism assumes equality concurrently with the higher importance of individual needs/drives; Singelis et al., 1995). Vertical individualistic behaviors have been associated with variations considering gaming patterns, such as goals and motivations, with more vertically orientated gamers being more attracted by competitive and achievement features, which likely exacerbate their susceptibility to IGD (Lee & Wohn, 2012; Stavropoulos, Anderson, et al., 2018; Stetina et al., 2011). In this context, the social and state functions in the USA present to be more “vertically” individualistic than those in Australia (Lee & Wohn, 2012; Singelis et al., 1995; Stavropoulos, Beard, et al., 2018). Subsequently, one could assume that more vertically individualistic players from the USA, might be at higher risk for IGD, when their gaming involvement is accompanied with high in-gaming achievements (provided within the MMO context), compared to their Australian counterparts (Beard et al., 2017). Interestingly, the sense of achievement (such as the sense of the in-game achievement could be viewed) is evaluated as more significant for the self-esteem of males than females (Bleidorn et al., 2016; Keller, Meier, Gross, & Semmer, 2013). This could in turn influence gaming contingence related drives differently for males and females across cultures, especially when they might present with inattention and hyperactivity-impulsivity that could vary in game performance (Anderson et al., 2017; Gomez et al., 2018).

### 1.6 The present study

This study conceptualized IGD behaviors upon a continuum from absence of behaviors to predominance (Pontes & Griffiths, 2014) to explore how MMO gamers' levels of different ADHD (i.e., inattentive and hyperactive/impulsive) symptoms may associate to IGD, taking into consideration gender differences. Furthermore, the present study aims to investigate whether these associations could vary across MMO gamers from the USA and Australia. The following hypotheses were defined:

H_1_ There will be a significant, positive association between inattentive symptoms and IGD features, regardless of country of origin.

H_2_ There will be a significant, positive association between hyperactive-impulsive and IGD features, regardless of country of origin.

H_3_ The relationship between ADHD type (hyperactive-impulsive or inattentive) and IGD will vary by gender.

H_4_ The IGD related effect of the interplay between genders with different types of ADHD symptoms could vary between USA and Australian gamers.

This exploratory hypothesis is supported by vertical individualism variations, which could interfere with the significance of achievement gaming-drives (Beard et al., 2017; Lee & Wohn, 2012; Singelis et al., 1995; Stavropoulos, Beard, et al., 2018; Stavropoulos, Burleigh, et al., 2018).

Finally, the potential prevalence of gaming disorder, as defined by scoring above the recommended cut-off of the Internet Gaming Disorder Scale-Short-Form (IGDS9-SF; Pontes & Griffiths, 2015) scale will be estimated for both samples of USA and Australian gamers. This effort is in line with the recommendation of the APA Substance-Related Disorders Work Group for more research on the gaming disorder prevalence (Hasin et al., 2013). Several studies have reported on the prevalence of disordered gaming among Australian and USA adolescents, with estimates varying from 4.4% and 9.4% respectively (Fam, 2018). However, these studies utilized a range of IGD assessment methodologies which make comparisons difficult. In contrast, the present study will use the same IGD assessment tool for both Australian and USA samples.

## 2 Method

### 2.1 Sample

The sample consisted of 163 Australian (M_age_ = 23.01, *SD* = 3.35, Min_age_ = 18, Max_age_ = 31, Males *n* = 121, 73.80%) and 457 U.S.-North American (M_age_ = 25.25 years, *SD* = 2.76, Min_age_ = 18 years, Max_age_ = 29 years, Males = 265, 57.98%). The estimated maximum sampling error regarding the 163 Australian gamers is 7.68%, for the 457 US gamers is 4.58% and for the 620 participants of the combined sample is 3.94% (Z = 1.96, confidence level 95%).

Considering the Australians, missing values ranged between 1% and 7% across all items and were determined to have been missing completely at random (Little's MCAR test *X*^*2*^ = 2142.904, *p* > .05; Little, 1988). The missing USA values ranged lower, between 0.2% and 4.8%, across all items and were also confirmed to have been missing completely at random (Little's MCAR test X2 = 8.35, DF = 7, p > .05; Little, 1988). Given that missing values were not systematic and in order not to reduce sample power (due to list-wise deletion), Maximum Likelihood Imputation was applied five times based on relevant literature (Stavropoulos, Anderson, et al., 2018).

### 2.2 Materials

Participants were asked to provide demographic (i.e. age, gender) and internet use (i.e. daily time of internet and gaming use on week days and the weekend) information before completing a battery of self-report questionnaires.

#### 2.2.1 Internet gaming disorder scale – short form 9 (IGDS9-SF)

The severity of IGD symptoms was measured using the IGDS9-SF, which reflects the nine IGD criteria (APA, 2013; Pontes & Griffiths, 2015). The scale has a total of nine items and employs a 5-point Likert scale: (1 = “Never” to 5 = “Very Often”), with total scores ranging from 9 to 45 (Pontes & Griffiths, 2015). Higher scores indicate higher IGD severity (Pontes & Griffiths, 2015). The instrument's internal reliability was high with a Cronbach's alpha = 0.92 for Australia and a Cronbach's alpha = 0.90 for USA gamers. A cut-off of 36 out of 45 has been suggested to distinguish between potentially disordered and non-disordered gamers (Pontes & Griffiths, 2015). This cut-off was used for both USA and Australian samples to address prevalence rates.

2.2. 2. Adult ADHD Self-Report Scale (ASRS-v1.1)

Adult ADHD symptoms were measured using the ASRS-v1.1 (Adler, Kessler, & Spencer, 2003). The instrument is divided into nine items assessing inattention (i.e. *how often do you make careless mistakes when you have to work on a boring or difficult project?*) and nine items assessing hyperactivity-impulsivity (i.e. *how often do you fidget or squirm with your hands or your feet when you have to sit down for a long time?*). All items are answered to 5-point Likert scale (1 = “Never” to 5 = “Very Often”), and total scores for the inattention (ranging between 9 and 45) and the hyperactivity-impulsivity (ranging between 9 and 45) subscales are produced by accumulating the relevant item points. The full ADHD scale score is produced based on the addition of the scores of the two subscales resulting a total score ranging from 18 to 90 points, with higher scores indicating higher ADHD symptoms. The internal reliability scores of the two subscales and the full scale were satisfactory for both samples (*Australian:* inattention Cronbach's alpha = 0.84, hyperactivity-impulsivity Cronbach's alpha = 0.86, ADHD Cronbach's alpha = 0.87; *USA:* inattention Cronbach's alpha = 0.92, hyperactivity-impulsivity Cronbach's alpha = 0.86, ADHD Cronbach's alpha = 0.95).

### 2.3 Procedure

The study was approved by the ethics committees of the Palo Alto University (USA) and Federation University (Australia). Eligible participants were MMO gamers, residents of Australia and the USA, aged between 18 and 29 years. The study was advertised online and data was collected via *SurveyMonkey* (Australia) and/or *Amazon Mechanical Turk* (USA; AMT). Participation was voluntary and participants were provided with a Plain Language Information Statement (PLIS) to consent before they engaged. They were free to withdraw at any time prior to submission of the survey. Online collection was preferred, as it provides higher accessibility and representation of internet gamers.

### 2.4 Statistical analyses and analytical strategy

To assess the association between inattention and IGD behaviors (H1), two (one for each country) hierarchical linear regression models (two steps) were conducted. To control for the confounding effects of gender (dummy coded 0 = females, 1 = males) and age on IGD behaviors (Anderson et al., 2017; Kuss, Griffiths, Karila, & Billieux, 2014), these were inserted separately as IGD predictors/confounders at step one. Inattention symptoms were then inserted at the second step. To assess the potential moderating/differentiating effect of the country of residence (Australia or USA) on this association, an additional moderation analysis was conducted on the combined sample using the PROCESS macro (Hayes, 2013). Inattentive symptoms were used as the independent variable, country of origin was used as the moderator (dummy coded 0 = Australia, 1 = USA), and IGD was used as the outcome variable (Hayes, 2013). Age and gender were inserted as covariates to address possible confounding effects (Hayes, 2013). Bootstrapping at the minimum recommended level of 1.000 resamples was applied (Bland & Altman, 2015).

Similarly, to assess the association between hyperactivity-impulsivity and IGD behaviors (H2), two more (one for each country) hierarchical linear regression models (two steps) were calculated. To control for the confounding effects of gender (dummy coded 0 = females, 1 = males) and age on IGD behaviors (Kuss et al., 2014), these were inserted separately at step one. Hyperactivity-impulsivity symptoms were then inserted (as an IGD predictor) at the second step. To assess the exploratory hypothesis considering the potential moderating/differentiating effect of the country of residence on this association, an additional moderation analysis was conducted on the combined sample using the PROCESS macro (Hayes, 2013). This employed the country of origin as the moderator (dummy coded 0 = Australia, 1 = USA). Age and gender were additionally controlled and bootstrapping at the minimum recommended level of 1.000 resamples was applied (Bland & Altman, 2015).

To assess H3, H4 referring to the likely moderating effect of gender on the association between hyperactivity-impulsivity and IGD behaviors and how this may differ between countries (exploratory hypothesis), a moderated moderation model was conducted using the PROCESS macro (Hayes, 2013). This model used the combined sample and inserted the country of origin (dummy coded 0 = Australia, 1 = USA) as a moderator of the moderating effect of gender (0 = Females, 1 = Males) on the association between hyperactivity-impulsivity and IGD behaviors. Age confounding effects were controlled and bootstrapping at 1.000 resamples was applied (Bland & Altman, 2015). A similar moderated moderation model was applied to address the interplay between inattention, IGD, gender and country (H3, H4)*,* with the only difference of using inattention symptoms as the independent variable. This aimed to assess whether there was a moderating effect of gender on the association between inattention and IGD and whether this differed between countries.

## 3 Results

Table 1 shows the descriptive measures for both USA and Australian samples. The estimated prevalence of IGD was higher for the USA sample (i.e., 3.9%; *N* = 18) compared to the Australian (i.e., 1.8%; *N* = 3). The USA sample also reported more ADHD symptoms compared to the Australian sample (44 ± 15.4 vs. 29.7 ± 10.7) for both inattention and hyperactivity-impulsivity.

**Table 1:** Descriptive statistics for both USA and Australian samples.

	USA			Australia		
	Total (n = 457)	Female (40%; 184/458)	Male (58%; 266/458)	Total (n = 164)	Female (24%; 40/164)	Male (74%; 121/164)
Age, years	25.3 ± 2.8	25.2 ± 2.5	25.3 ± 2.9	23.0 ± 3.4	22.9 ± 3.3	23.0 ± 3.4
IGDS9-SF: total score	20.6 ± 7.8	19.8 ± 7.7	21.3 ± 7.7	19.2 ± 6.9	20.3 ± 8.4	18.7 ± 6.2
IGDS9-SF: disordered (≥36)	3.9% (18/436)	2.2% (4/176)	4.9% (13/252)	1.8% (3/164)	7.5% (3/40)	0% (0/121)
ASRS: total score	44.0 ± 15.4	41.7 ± 15.9	45.5 ± 14.9	29.7 ± 9.5	29.2 ± 10.7	29.8 ± 9.2
ASRS: inattention total	22.8 ± 8.3	21.8 ± 8.8	23.5 ± 8.0	16.6 ± 4.2	16.3 ± 4.4	16.7 ± 4.2
ASRS: hyperactivity-impulsivity total	21.3 ± 7.7	20.0 ± 7.9	22.1 ± 7.4	13.7 ± 5.1	13.6 ± 5.8	13.7 ± 5.0

IGDS9-SF = Internet Gaming Disorder Scale – Short Form 9; ASRS = Adult ADHD Self-Report Scale.

Considering H1 in the USA sample_*,*_ the slope of the regression line was statistically significant (*F*_(2,413__)_ = 122.69, *p* < .001). Specifically, 22.9% of the IGD variance was explained by inattentive ADHD (*R*^*2*^ = 0.229). For each point of increase in inattention, IGD increased by 0.44 (*b* = 0.44, *t =* 10.96, *p* < .001). The Australian sample results showed similar results, with a statistically significant regression slope (*F*_(3,160__)_ = 13.36, *p* = .001). Specifically, 7.6% of the IGD variance was explained by inattention (*R*^*2*^ = 0.076). For each point of increase in inattention, IGD scores increased by 0.45 (*b* = 0.45 *t =* 3.655, *p* = .001). To assess the potentially moderating effect of country on the association between Inattention and IGD behaviors the following model was additionally estimated:

$$\mathrm{IGD}\;\text{score}=a+\text{b1 inattention scores}+\text{b2 country}+\text{b3}\;\left[\text{inattention scores}\times \text{country}\right]+\text{b4}\;\mathrm{age}+\text{b5 gender}.$$

Findings indicated that country [*b2* = 1.05, *t* = 0.44, *p* = .659, *LLCI* = −3.60 – *ULCI* = 5.69] and the interplay between inattention and country [*b3* = 0.01, *t* = 0.07, *p* = .945, *LLCI* = −0.249 – *ULC I* = 0.268] on IGD were not statistically significant, with the latter explaining <0.01% of the IGD score variance (*R*^*2*^ = 0.000).

Considering H2 for the USA, results demonstrated that the regression slope was statistically significant (F _*(3,405)*_ = 55.84, *p* < .001). In particular, 29.3% of the IGD variance was explained by hyperactivity-impulsivity (*R*^*2*^ = 0.293). For each point of increase of hyperactivity-impulsivity, IGD increased by 0.56 (*b* = 0.55, *t =* 12.805, *p* = .001). Similarly, for the Australian sample, the regression slope was statistically significant (F _*(3,160)*_ = 5.88, *p* < .001). In particular, 9.9% of the IGD variance was exclusively explained by hyperactivity-impulsivity (*R*^*2*^ = 0.099). For each point of hyperactivity-impulsivity increase, IGD scores increased by 0.35 (*b* = 0.35, *t =* 3.50, *p* = .001). To assess the likely moderating effect of country on this association the following model was estimated:

$$\mathrm{IGD}\;\text{score}=a+\text{b1 hyperactivity impulsivity scores}+\text{b2 country}+\text{b3}\;\left[\text{hyperactivity impulsivity scores}\times \text{country}\right]+\text{b4}\;\mathrm{age}+\text{b5 gender}.$$

The interaction between hyperactivity-impulsivity and country [*b3* = −0.18, *t* = 1.68, *p* = .093, *LLCI* = −0.398 – *ULCI* = 0.031] on IGD was insignificant explaining <1% of the IGD variance (*R*^*2*^ = 0.004).

To address H3, H4 in regards to hyperactivity a moderated moderation analysis was conducted adopting the methodology recommended by Hayes (2013). The model estimated was the following:

$$\mathrm{IGD}=a+\text{b1 hyperactivity}+\text{b2 Gender T2}+\text{b3 Country}+b4\mathrm{age}+b5\left[\text{hyperactivity}\times \text{gender}\right]+b6\left[\text{hyperactivity}\times \text{country}\right]+b7\left[\text{country}\times \text{gender}\right]+b8\left[\text{hyperactivity}\times \text{gender}\times \text{country}\right];$$

**Fig. 1:**
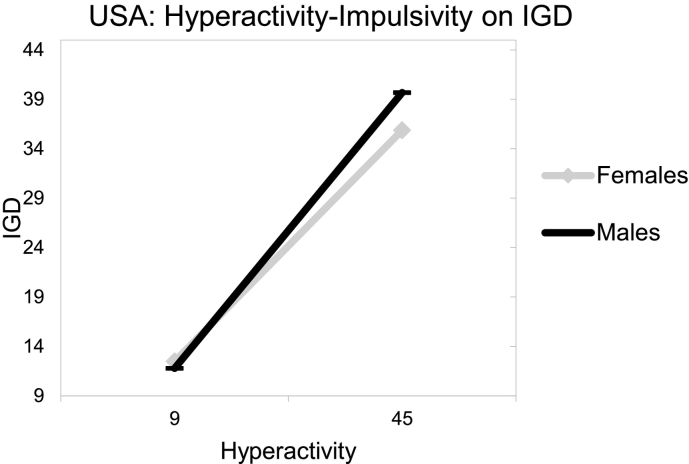
USA: hyperactivity-impulsivity on IGD.

**Fig. 2:**
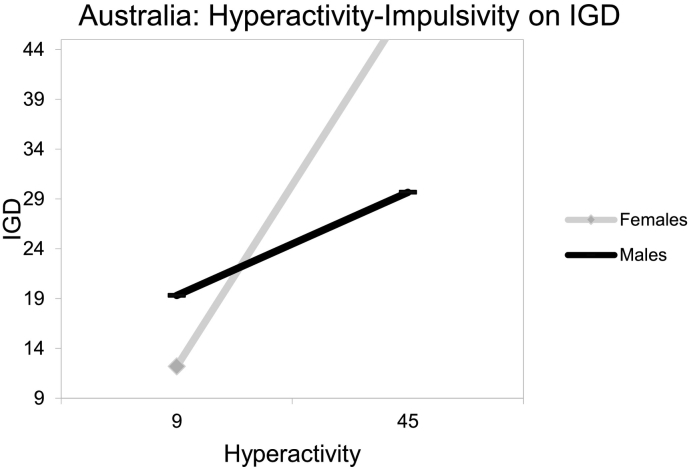
Australia: hyperactivity-impulsivity on IGD.

The full model accounted for 26.18% of IGD variance. The regression slope for the overall model was significant (*F*_*(8, 564)*_ = 25.00, *p* < .001; see Table 2). An inspection of the two-way interaction coefficient between country and gender indicated that they significantly interacted (risk effect for USA males) considering IGD (*b8* = 7.83, *t* = 2.27, *p* = .024). Similarly, an inspection of the three-way interaction coefficient between hyperactivity-impulsivity, country and gender indicated a significant protective interaction considering IGD (*b8* = − 0.68, *t* = −3.03, *p* = .002). Specifically, when an MMO gamer presented concurrently with a higher point (than the intercept) in hyperactivity-impulsivity, was male and Australian, then IGD reduced by 0.68 (see Fig. 1, Fig. 2).

**Table 2:** Estimating IGD behaviors from hyperactivity-impulsivity symptoms, gender, country and their interactions.

	b	SE	t	p	LLCI	UCLI
α: Constant/intercept	12.48	2.76	4.53	0.000	7.07	17.89
b1: Hyperactivity (F)	0.52	0.06	9.43	0.000	0.41	0.63
b2: Gender (M_1_)	−0.69	1.22	−0.56	0.573	−3.09	1.71
b3: Country (M_2_) b4: Age b5: Hyperactivity × gender	−0.29	2.97	−0.10	0.921	−6.14	5 0.55
	−0.12	0.09	−1.31	0.191	−0.31	0.06
	0.03	0.05	0.58	0.565	−0.07	0.13
b6: Hyperactivity × country	0.29	0.19	1.54	0.123	−0.08	0.67
b7: Country × gender	7.83	3.45	2.27	0.024	1.06	14.60
b8:Hyperactivity × gender × country	−0.68	0.23	- 3.03	0.003	−1.13	−0.24

Note. *b =* estimated value of unstandardized regression coefficient; *SE* = standard error; *t* = *t*-test statistic; *p* = probability; *LLCI* = lower level confidence interval; *UCLI* = upper level confidence interval.

To address H3, H4 considering inattention, a similar moderated moderation analysis was conducted for the model below:

$$\mathrm{IGD}=a+\text{b1 inattention T1}+\text{b2 Gender T2}+\text{b3 Country}+b4\mathrm{age}+b5\left[\text{inattention}\times \text{gender}\right]+b6\left[\text{inattention}\times \text{country}\right]+b7\left[\text{country}\times \text{gender}\right]+b8\left[\text{inattention}\times \text{gender}\times \text{country}\right];$$

**Fig. 3:**
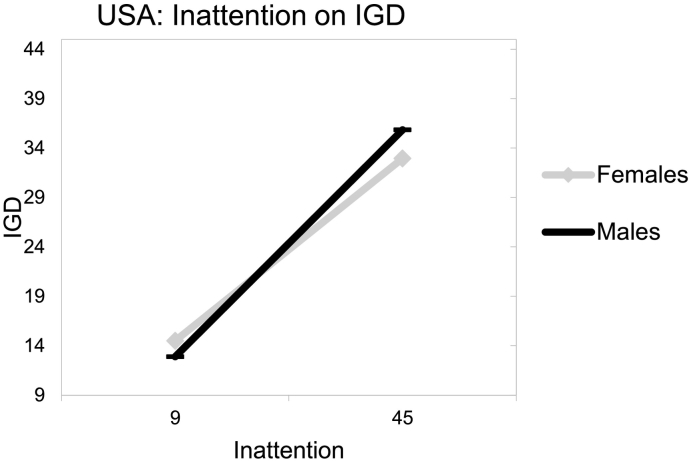
USA: inattention on IGD.

**Fig. 4:**
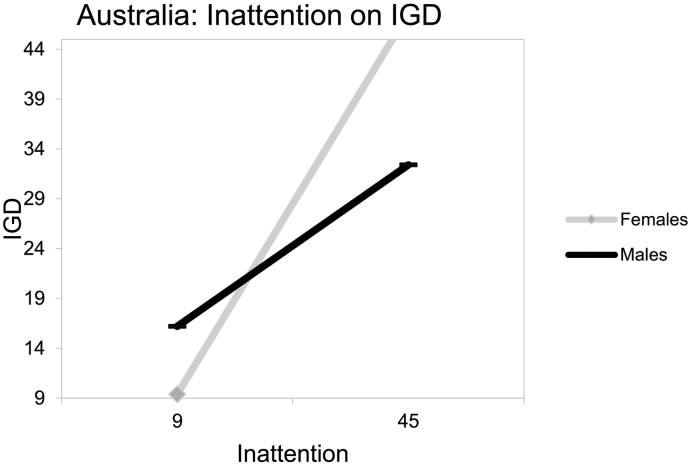
Australia: inattention on IGD.

The full model accounted for 20.93% of IGD variance. The regression slope for the overall model was significant (*F*_*(8, 571)*_ = 18.89, *p* < .001; see Table 3). An inspection of the three-way interaction coefficient between inattention, country and gender indicated a significant protective interaction considering IGD (*b* = − 0.59, *t* = −2.00, *p* = .046). Specifically, when an MMO gamer presented concurrently with a higher point (than the intercept) in inattention, was male and Australian, then IGD reduced by −0.59 (see Fig. 3, Fig. 4).

**Table 3:** Estimating IGD behaviors from inattention symptoms, gender, country and their interactions.

	b	SE	t	p	LLCI	UCLI
α: Constant/intercept	14.51	2.84	5.11	0.000	8.93	20.09
b1: Inattention (F)	0.41	0.05	7.71	0.000	0.30	0.51
b2: Gender (M_1_)	−1.61	1.45	−1.11	0.268	−4.45	1.24
b3: Country (M_2_) b4: Age b5: Inattention × gender	−5.12	4.36	−1.17	0.241	−13.68	3.44
	−0.12	0.10	−1.23	0.220	−0.31	0.07
	0.05	0.06	0.93	0.350	−0.06	0.16
b6: Inattention × country	0.44	0.25	1.76	0.080	−0.05	0.94
b7: Country × gender	8.42	5.10	1.66	0.098	−1.56	18.41
b8:Inattention × gender × country	−0.59	0.29	- 2.00	0.046	−1.16	−0.01

Note. *b =* estimated value of unstandardized regression coefficient; *SE* = standard error; *t* = *t*-test statistic; *p* = probability; *LLCI* = lower level confidence interval; *UCLI* = upper level confidence interval.

Conclusively, findings indicated that both hyperactivity and inattention associated with higher IGD risk among emergent adult, MMO gamers independent of the country of origin. Furthermore, there seemed to be gender and country variations with males from USA presenting with ADHD symptoms being at higher IGD risk than their Australian counterparts.

## 4 Discussion

The present study independently examined the associations between inattention and hyperactivity-impulsivity symptoms and IGD risk, gender, and country of residence, in a combined sample of Australian and USA emergent adult MMO players. Hierarchical linear regression, moderation and moderated moderation analyses confirmed that higher inattention and hyperactivity-impulsivity symptoms were interwoven with higher IGD behaviors across both national samples. Interestingly, the intensity of these associations was revealed to differentiate based on their interplay with the gender of the gamer and his/her country of origin (i.e., Australia or USA). Specifically, more hyperactive-impulsive, as well as inattentive males in the USA presented higher on IGD behaviors than their Australian counterparts. These results reinforced past literature hypotheses considering culturally related fluctuations of the effects of IGD risk factors. Furthermore, findings highlighted that such variations need to be taken into account when studying individual IGD risk effects, such as gender and the reported levels of other concurrent psychopathology. Finally, our data indicated that nearly 4% of USA gamers and nearly 2% of Australian gamers are potentially at risk for disordered gaming. While these proportions are lower than that previously reported (Fam, 2018), they do follow the trend of higher IGD prevalence among USA adolescents when compared to Australian. Nevertheless, due to the relatively smaller sample sizes reported here, these estimations should be treated with caution.

### 4.1 Inattentive ADHD and IGD

The study envisaged and confirmed that MMO gamers experiencing higher inattention symptoms would report elevated IGD behaviors (H1), and this would be consistent across USA and Australian gamers. This assumption aligns with a series of both cross-sectional and longitudinal findings illustrating the association of inattention and; a) addictive behaviors in general (Harstad et al., 2014); b) behavioral addictions (Allami et al., 2017) and; c) technological, internet and gaming related dependency behaviors in particular across various populations (Anderson et al., 2017; Wartberg, Kriston, Zieglmeier, Lincoln, & Kammerl, 2018). Studies have suggested that the link between inattention and IGD could be potentially explained due to the immediate response and reward system that MMO gaming provides (Yen et al., 2009). This may in turn alleviate the boredom, which is typically reported by inattentive users, and potentially concurrently introduce lack of responsivity to real world rewards (Yen et al., 2009). Furthermore, gaming could offer an escape from the real-life problems that a gamer could be experiencing as a result of inattention (Weinstein & Weizman, 2012). Nevertheless, it should be considered that the association between inattention and IGD symptoms may not be unidirectional but bi-directional, with inattentive symptoms precipitating IGD presentations, which in turn may perpetuate pre-existing inattention (Stavropoulos, Beard, et al., 2018; Stavropoulos, Burleigh, et al., 2018; Vadlin et al., 2016). Despite the above, the present finding may expand the knowledge in the field in two ways. First, inattention was examined separately to hyperactivity, and therefore the confounding effects of other forms of ADHD symptoms in its association with IGD were accounted for. Second, due to the dearth of past relative cross-cultural examinations, the finding considering the lack of significant differences in the inattention-IGD association between Australian and USA gamers is (to the best of the authors' knowledge) innovative. This is significant, as it indicates that the risk effect of inattention could overarch cultural differences, and therefore should be carefully considered in treatment protocols applied worldwide. Nevertheless, studies across more diverse cultural populations are recommended.

### 4.2 Hyperactive/impulsive ADHD and IGD

Similarly, the hypothesis (H2) that MMO gamers with higher hyperactivity-impulsivity symptoms would present with higher IGD behaviors was supported. In line with the hypothesis one finding, there was no significant differences between Australian and USA gamers considering this association. This finding converges with past literature which demonstrated that gamers with higher levels of impulsivity are at higher risk of developing IGD symptoms (Ding et al., 2014; Rikkers et al., 2016; Yen et al., 2017). Past hypothesized explanations suggested that the relationship between hyperactivity-impulsivity and IGD symptoms may be due to the impulse control deficits shared by the two clinical presentations (Ding et al., 2014; Yen et al., 2017). Such control deficits could be attenuated by the immediate reward systems integrated within the context of internet games. These could reinforce the drives for positive immediate gratification, which are interrelated with addiction presentations such as IGD (Anderson et al., 2017).

Overall, the present study's results serves to increase the current knowledge surrounding the association between hyperactivity-impulsivity and IGD symptoms in two significant ways. First, the present study differentiates the effect of hyperactivity-impulsivity from inattention symptoms on IGD behaviors. Second, as with the case of inattention related IGD effects, it indicated that this effect exhibited cross-cultural resilience across groups of Australian and USA gamers. Nevertheless, as mentioned earlier studies across more diverse cultures are needed. Furthermore, in order to assess *development* of IGD, longitudinal studies must be conducted to answer important empirical questions about the ADHD-IGD temporal association.

### 4.3 Hyperactivity-impulsivity, inattention and IGD: The interplay of gender and country

The present study's hypotheses (H3, H4) that male MMO gamers with higher hyperactivity-impulsivity symptoms, as well as higher inattention symptoms, would present with higher IGD behaviors and this could vary between USA and Australian gamers was supported. Based on previous literature, the increased IGD risk of hyperactive-impulsive and inattentive males could be attributed to their higher engagement with internet gaming, which is typically perceived as a more masculine activity (Anderson et al., 2017). This potential explanation is further reinforced by findings supporting that: a) the game content tends to be more directed at males and; b) that females tend to play for shorter amounts of time, to experience lower levels of online flow and to play fewer games than males (Bryce & Rutter, 2003; Stavropoulos et al., 2013b).

In that line, the variations of the hyperactivity-impulsivity, as well as inattention symptoms and IGD behaviors associations revealed here, consistently to the detriment of USA gamers appear to also align with past literature (Pontes et al., 2017). Culture has been suggested to be significantly involved with aspects of addictions (e.g., prevalence, incidence, clinical manifestations), potentially increasing an individual's susceptibility, as well as fluctuating the intensity of associated risk effects, such as hyperactivity-impulsivity or gender (Landrine et al., 1992; Stavropoulos, Anderson, et al., 2018; Wilsnack et al., 2000). Studies suggest that vulnerability differences to specific forms of psychopathology, like that of IGD, could be effected by “vertical” and “horizontal” individualism (Clemens et al., 2014; Singelis et al., 1995; Stavropoulos et al., 2013a; Stavropoulos, Anderson, et al., 2018; Stavropoulos, Griffiths, et al., 2018). Vertical individualism, as a cultural characteristic, emphasizes personal achievements and competition to determine social values and is assumed to be higher in the USA than Australia (Singelis et al., 1995; Stavropoulos, Griffiths, et al., 2018). Therefore, and in relation to gaming in particular, it could be assumed that in game achievements would exacerbate gaming involvement (and thus IGD risk) more for hyperactive-impulsive, as well as inattentive, male, USA gamers for a series of reasons. Improved game performance would be expected to be valued as more significant for the self-esteem of males (than females) and for more vertically individualistic USA gamers, due to their higher proneness to achievement and competitive drives (Abikoff et al., 2002; Anderson et al., 2017; Beard et al., 2017; Biederman et al., 2002; Bozkurt et al., 2013; Hyun et al., 2015; Lee & Wohn, 2012; Singelis et al., 1995; Stavropoulos et al., 2013a; Stavropoulos, Anderson, et al., 2018; Stavropoulos, Griffiths, et al., 2018; Yen et al., 2009). These are significant, and to the best of the authors' knowledge, they constitute the first relevant findings separately examining: a) the interplay between hyperactivity-impulsivity symptoms and gender and; b) the interplay between inattention symptoms and gender, across different countries. Specifically, the need for both research and clinical initiatives targeting IGD to be more sensitive to the interplay between gender and cultural differences is reinforced by these findings. In that line, further research distinctly examining the longitudinal associations/interplay between hyperactivity-impulsivity, as well as inattentive symptoms, gender and IGD across different cultures should be invited given the global nature of the disorder.

### 4.4 Conclusions, limitations and future research

The importance of these results can be reflected in their ability to identify the differential impact that the interplay between gender and culture may introduce on the associations between the two different types of ADHD symptoms and IGD. Specifically, emphasis on the higher IGD risk of more vertically individualistic male players, presenting with either inattention or/and hyperactivity-impulsivity symptoms should be taken into consideration when planning relevant prevention and treatment interventions worldwide.

Nevertheless, the present study has significant limitations. Among others, these include the use of self-report questionnaires, where participants' ability to accurately estimate their gaming time is questionable (see Alexandraki et al., 2018; Kahn, Ratan, & Williams, 2014). Alongside this, due to the cross-sectional nature of the study, the direction of causality can only be hypothesized on the basis of theoretical arguments. Finally, the present study utilized participants limited to Australian and USA citizens/residents, therefore the cultural variations identified may not be equally generalizable across other cultures. Related to this point, was the higher rate of ADHD symptomatology that was self-reported in the USA compare to the Australian sample. Future work should explore whether the associations reported here are still present with comparable rates of ADHD symptomatology. Future research should look to utilizing longitudinal designs, embracing more objective measures (e.g. monitored game playing time), to potentially better identify direct causal inferences between the studied constructs. Furthermore, such research should involve more diverse culturally populations of gamers, as well as objective measurements of individualism-collectivism.

## Conflict of interest

The authors report no conflicts of interest.
